# Spatial-temporal excess mortality patterns of the 1918–1919 influenza pandemic in Spain

**DOI:** 10.1186/1471-2334-14-371

**Published:** 2014-07-05

**Authors:** Gerardo Chowell, Anton Erkoreka, Cécile Viboud, Beatriz Echeverri-Dávila

**Affiliations:** 1 Division of International Epidemiology and Population Studies, Fogarty International Center, National Institutes of Health, Bethesda, MD, USA; 2 Mathematical, Computational & Modeling Sciences Center, School of Human Evolution and Social Change, Arizona State University, Tempe, AZ, USA; 3 Basque Museum of the History of Medicine and Science. University of the Basque Country, Bilbao, Spain; 4 Grupo de Estudios de Población y Sociedad, Universidad Complutense de Madrid, Madrid, Spain

**Keywords:** 1918–1919 influenza pandemic, Spain, Spanish influenza, Spring-summer wave, Excess death rates, Relative risk of death, Transmissibility, Provinces, Geography, Spatial heterogeneity

## Abstract

**Background:**

The impact of socio-demographic factors and baseline health on the mortality burden of seasonal and pandemic influenza remains debated. Here we analyzed the spatial-temporal mortality patterns of the 1918 influenza pandemic in Spain, one of the countries of Europe that experienced the highest mortality burden.

**Methods:**

We analyzed monthly death rates from respiratory diseases and all-causes across 49 provinces of Spain, including the Canary and Balearic Islands, during the period January-1915 to June-1919. We estimated the influenza-related excess death rates and risk of death relative to baseline mortality by pandemic wave and province. We then explored the association between pandemic excess mortality rates and health and socio-demographic factors, which included population size and age structure, population density, infant mortality rates, baseline death rates, and urbanization.

**Results:**

Our analysis revealed high geographic heterogeneity in pandemic mortality impact. We identified 3 pandemic waves of varying timing and intensity covering the period from Jan-1918 to Jun-1919, with the highest pandemic-related excess mortality rates occurring during the months of October-November 1918 across all Spanish provinces. Cumulative excess mortality rates followed a south–north gradient after controlling for demographic factors, with the North experiencing highest excess mortality rates. A model that included latitude, population density, and the proportion of children living in provinces explained about 40% of the geographic variability in cumulative excess death rates during 1918–19, but different factors explained mortality variation in each wave.

**Conclusions:**

A substantial fraction of the variability in excess mortality rates across Spanish provinces remained unexplained, which suggests that other unidentified factors such as comorbidities, climate and background immunity may have affected the 1918–19 pandemic mortality rates. Further archeo-epidemiological research should concentrate on identifying settings with combined availability of local historical mortality records and information on the prevalence of underlying risk factors, or patient-level clinical data, to further clarify the drivers of 1918 pandemic influenza mortality.

## Background

The “Spanish Influenza” pandemic is the infectious disease event associated with the highest mortality burden in recent history, with global mortality burden estimates ranging from 20 to 50 million deaths [1,2]. The pandemic was coined “Spanish Influenza” because the Spanish press widely publicized the outbreak in its early stages, as significant increases in respiratory mortality were reported in several Spanish provinces during May-June 1918 [3,4]. In contrast, the rest of Europe censored all news relating to the pandemic for fear of a decline in troop morale in the midst of World War I.

A characteristic feature of the 1918 influenza pandemic is the disproportionate increase in mortality rates among young adults relative to pre-pandemic years, consistent across populations with different geographic, demographic, and socio-economic background [5-13]. Lung tissue sections obtained from archived autopsy material indicate that most influenza-related fatalities in 1918 were associated with secondary bacterial pneumonia [14,15] while in contrast children seldom developed fatal bacterial pneumonia [16]. Another notable feature of the 1918 pandemic is the multiple wave profile of infection that included the sporadic occurrence of mild herald waves in spring and summer 1918 [13]. Moreover, reports from North America and Europe have underscored significant sparing of senior populations during the main fall 1918 pandemic wave, a phenomenon that likely resulted from prior immunity acquired from childhood exposure to related influenza viruses [7]. In contrast, senior populations suffered significant pandemic death rates in Mexico [7], Colombia [8] and remote populations [17]. Hence, geographic differences in the age-specific mortality rates of the 1918 pandemic may in part originate from differences in background immunity, shaped by heterogeneous circulation of influenza viruses prior to the 1918 pandemic [7].

In Europe, the excess mortality rate associated with the 1918–19 influenza pandemic has been estimated at 1.1%, representing an 86% elevation of all-cause mortality relative to background death rate in non-pandemic periods [18]. The highest relative risk of death in Europe has been reported in Italy (172%) followed by Bulgaria and Portugal (102%) and Spain (87%) while the lowest relative risk of death was observed in Finland (33%) [18]. Mortality rates peaked in the months of October-November 1918 in Europe, with southern countries experiencing significantly higher excess mortality rates than northern countries [18]. Moreover, a herald pandemic wave was reported in spring and summer 1918 in Spain, Portugal, Germany, Bulgaria, Switzerland, Finland and Denmark [18,19].

The reasons behind the large geographical variations in timing and mortality burden of 1918–1920 influenza pandemic waves remain debated [1,20]. Analyses of pandemic mortality impact at refined spatial and temporal scales together with geographic, demographic, and socioeconomic data can help shed light on the putative drivers of pandemic mortality. Such studies can in turn inform pandemic preparedness efforts by identifying subpopulations at elevated risk of influenza mortality, which could be prioritized in the case of limited vaccines or treatments. However, there are been few spatial-temporal analyses of the impact of the 1918 influenza pandemic at a subnational scale [21-26]. Here we report on the mortality patterns of the 1918 influenza pandemic across Spain, a country where few quantitative reports of excess mortality rates exist [3,27-29]. We modeled monthly mortality statistics across 49 Spanish provinces including the Canary and Balearic Islands covering January-1915 to June-1919 to quantify geographic and temporal patterns in excess death rates and relative risk of death during the pandemic period. We then explored the association of pandemic mortality patterns with demographic and socio-economic factors.

## Methods

### Data sources

#### Mortality statistics, Spain, 1915–1919

We compiled monthly all-cause and respiratory mortality statistics from January 1915 to June 1919 across 49 provinces of Spain including the Canary and Balearic Islands [30]. We used data starting in 1915 to estimate a robust mortality baseline for non-pandemic years and quantify the excess contribution of pandemic influenza in 1918–1919. Respiratory mortality comprised pneumonia, bronchopneumonia, influenza, bronchitis, and all other respiratory causes but for tuberculosis.

#### Demographic variables

We obtained 1915 population estimates [30] to calculate death rates and compiled latitude and longitude coordinates of the capital city of each province to explore pandemic timing across provinces. We also retrieved infant mortality rates [31] as a proxy for health index, and created an urbanization index (defined as the proportion of the population living in the capital of each province in 1915) [32]. We also compiled the population density [33], and the 1920 age-stratified population size [32] in order to estimate the proportion of children aged 5–15 years by province, as school-age children are thought to drive influenza transmission. The socio-demographic variables used in our analysis are summarized in Table 1.

**Table 1:** **The range, median and interquartile range of socio-demographic variables in 49 provinces of Spain used in our analyses **[30-33]

**Variable**	**Range**			**Interquartile range**	
	**Minimum**	**Maximum**	**Median**	**Lower bound**	**Upper bound**
**Population size**	97,956	1,176,044	368,024	277,698	510,315
**Population density (/km**^**2**^**)**	14.7	189.1	40.8	25.7	64.1
**Proportion of children, 5–15 y (%)**	26.4	35.9	33.2	31.3	34.0
**Infant mortality rates per 100,000 (<1 year)**	156.3	772.3	472.2	342.9	618.2
**Urbanization (%)**	4.0	68.2	9.4	5.7	20.8

### Statistical analysis

#### Estimation of excess pandemic mortality attributable to influenza

To quantify the mortality burden associated with the 1918–1919 influenza pandemic and explore the timing of the pandemic waves, we defined a discrete period of pandemic influenza activity, and estimated mortality occurring in excess of background deaths during the pandemic period. Because mortality levels tend to oscillate seasonally throughout the year during this time period in Spain, our background mortality estimate must also vary seasonally. To estimate baseline mortality in the absence of influenza activity, we fit cyclical regression models to monthly pre-pandemic mortality data for 1915–17, including temporal trends and harmonic terms for seasonality [6,19,34,35]. Periods of significant mortality elevation over the model baseline are indicative of influenza activity.

Pandemic periods were defined separately for each province as the months when observed mortality levels exceeded the upper limit of the 95% confidence interval of the baseline model. We then summed the excess deaths above the model baseline during each pandemic period identified during 1918–19 to estimate pandemic burden.

We also calculated the relative risk of pandemic death, defined as the ratio of the excess mortality during the pandemic periods to the expected baseline mortality for these periods. The relative risk measure has been shown to facilitate comparison between countries, regions, or age groups, which have different background risks of deaths [35,36].

### Geographic patterns

For each Spanish province, we estimated the peak timing of pandemic mortality defined as the month with maximal mortality elevation during the pandemic period. We also explored the association between province-level estimates of peak timing, excess death rate, and relative risk with latitude, population size, population density, proportion of children in population, urbanization index, and infant mortality rates. Finally, we built a multivariate linear regression model with all predictor variables to disentangle the factors explaining geographical variation in absolute and relative pandemic death rates. We generated parsimonious models by means of backward stepwise elimination.

### Spatial autocorrelation

We also quantified the extent of spatial autocorrelation in mortality data across the 49 Spanish provinces using Moran’s I statistic [33] with a nearest-neighbor spatial mixing matrix [37]. We applied the test to cumulative excess mortality rates and relative risk ratios for the period 1918–1919. We assessed statistical significance via randomization by generating an empirical null distribution (no-auto-correlation) based on 10,000 permutations of the regional assignment in original data [34].

## Results

**Figure 1:**
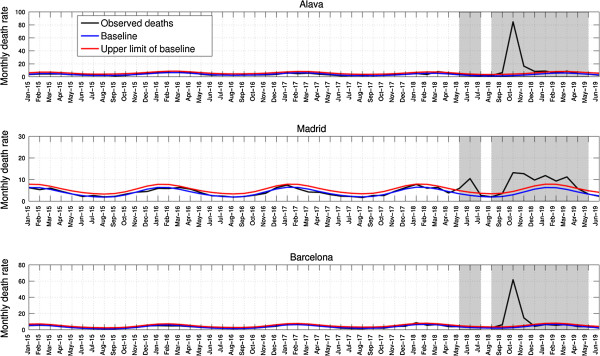
**Monthly respiratory deaths per 10,000 people in three representative provinces of Spain, Jan-1915 to June-1919.** The black curve is the monthly number of respiratory deaths. Shaded areas highlight time periods of high mortality associated with the 1918–1919 pandemic in Spain. The Serfling seasonal regression model baseline (blue curve) and corresponding upper limit of the 95% confidence interval of the baseline (red curve) are also shown. Excess deaths are above the upper limit of the baseline mortality curve calibrated using mortality levels prior to the 1918 influenza pandemic.

**Figure 2:**
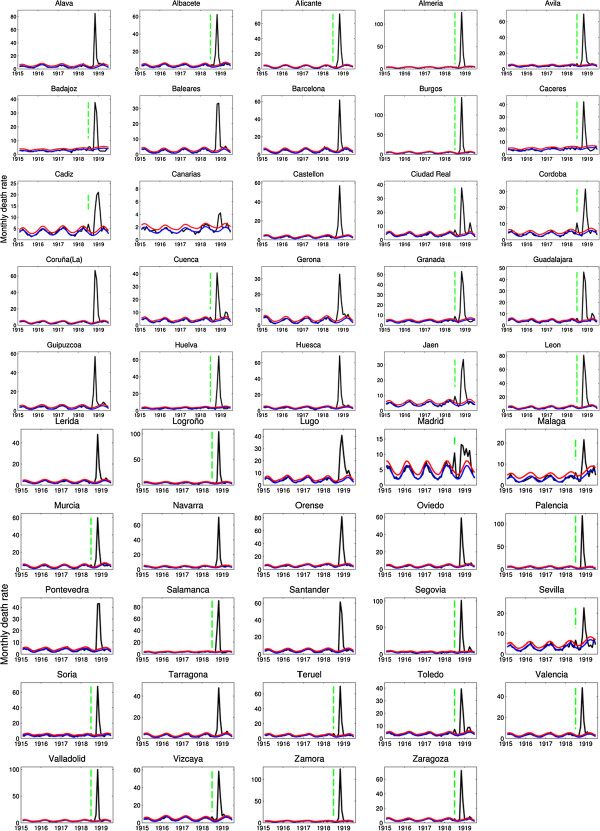
**Monthly respiratory deaths per 10,000 people in 49 provinces of Spain, Jan-1915 to June-1919.** The black curve is the monthly number of respiratory deaths. Vertical green dashed lines indicate the presence of summer mortality waves whenever respiratory mortality rates exceeded the seasonal mortality baseline in any spring-summer month. The Serfling seasonal regression model baseline (blue curve) and corresponding upper limit of the 95% confidence interval of the baseline (red curve) are also shown. Excess deaths are above the upper limit of the baseline mortality curve calibrated using mortality levels prior to the 1918 influenza pandemic. Individual figures display different scales in the Y-axis.

**Figure 3:**
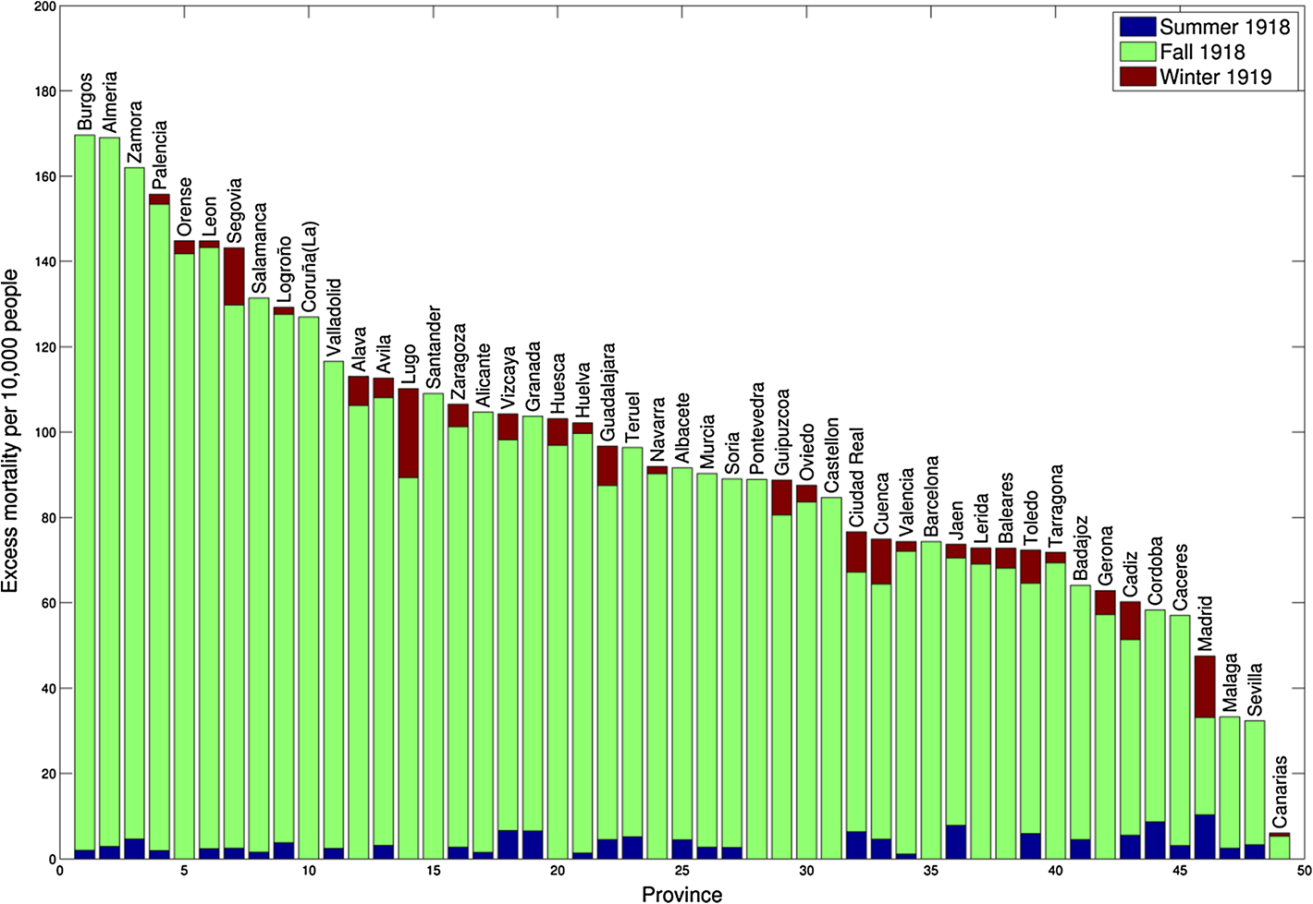
**Excess respiratory mortality rates per 10,000 across 49 provinces of Spain.** Results are shown for three pandemic periods (May-July 1918, August 1918-December 1918, and January 1919-April 1919) and sorted from high to low excess death rates. Excess deaths are above the upper limit of the baseline mortality curve calibrated using respiratory monthly mortality levels prior to the 1918 influenza pandemic.

**Figure 4:**
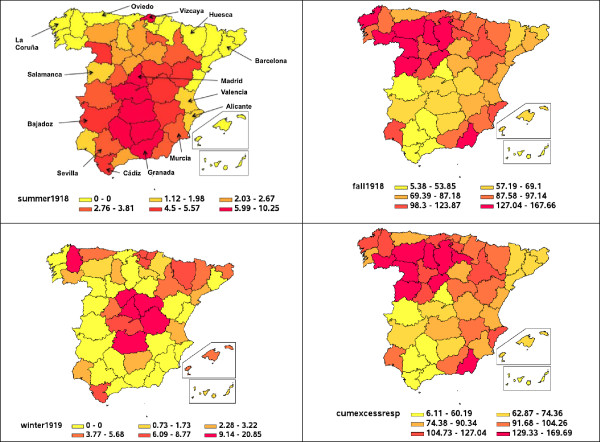
**Maps of excess respiratory deaths rates per 10,000 across provinces of Spain.** Maps are shown for three pandemic periods corresponding to spring (May 1918-July 1918), fall (August 1918-December 1918), and winter (January 1919-April 1919) pandemic waves and the cumulative excess respiratory death rate associated with the 1918–1919 influenza pandemic.

Spain experienced 3 pandemic mortality waves in spring-summer 1918, fall 1918 and winter 1919 (Figure 1). Our results indicate that the first pandemic wave in May-July 1918 generated relatively mild excess mortality rates among affected provinces (Figure 2). The mean excess respiratory mortality rate estimate for the first pandemic wave was 2.4 per 10,000 (range across provinces, 0–10.3 per 10,000); excess mortality estimates from all-cause and respiratory causes were well correlated (Spearman rho = 0.72, P < 0.001). We identified significant excess respiratory death rates in 32 of the 49 provinces during the spring-summer 1918, with Madrid experiencing the highest excess respiratory death rate estimated at 10.3 per 10,000 (Table 2, Figure 3) or a 1.68-fold increase over baseline respiratory mortality rate during this period (Additional file 1: Figure S1). Other provinces with high excess mortality rates during the 1918 spring-summer wave include the central provinces of Toledo and Ciudad Real and extending south to Cordoba, Jaen, and Granada (Figure 4). The provinces of Vizcaya in north Spain and Cadiz in the south were also significantly affected by this pandemic wave (Figure 4). By contrast, the Canary and Balearic Islands did not experience excess mortality during this early wave (Table 2). Provinces with higher baseline death rates and urbanization indices experienced higher excess respiratory death rates during the spring-summer 1918 wave and these two factors explained 78% of the variance in pandemic burden (P < 0.0001, Table 3). We also detected significant spatial autocorrelation in excess respiratory death rates during the spring-summer wave (Moran’s I test, P = 0.004).

**Table 2:** Estimates of excess mortality rates attributable to pandemic influenza based on respiratory and all-cause mortality rates across 49 provinces of Spain

**Province**	**Respiratory mortality**			**All-cause mortality**		
	**May-July 1918**	**August 1918- April 1919**	**Cumulative excess mortality 1918-1919**	**May-July 1918**	**August 1918- April 1919**	**Cumulative excess mortality 1918-1919**
Burgos	2.0	167.7	169.7	0.0	212	212
Almeria	2.9	166.2	169.1	0.0	187.1	187.1
Zamora	4.7	157.4	162.1	8.9	203.5	212.4
Palencia	2.0	153.7	155.7	6.3	184.1	190.4
Orense	0.0	144.9	144.9	0.0	171.6	171.6
Leon	2.4	142.5	144.9	3.8	176	179.8
Segovia	2.5	140.6	143.1	0.0	154.7	154.7
Salamanca	1.6	129.9	131.5	0.0	163.2	163.2
Logroño	3.8	125.5	129.3	0.0	156.1	156.1
Coruña (La)	0.0	127	127.0	0.0	142.5	142.5
Valladolid	2.5	114.1	116.6	4.5	151.3	155.8
Alava	0.0	113.1	113.1	0.0	135.5	135.5
Avila	3.2	109.5	112.7	0.0	153.9	153.9
Lugo	0.0	110.2	110.2	0.0	129.9	129.9
Santander	0.0	109.1	109.1	0.0	121.8	121.8
Zaragoza	2.8	103.8	106.6	0.0	122.5	122.5
Alicante	1.6	103.2	104.8	0.0	116.9	116.9
Vizcaya	6.7	97.6	104.3	8.5	117.4	125.9
Granada	6.6	97.1	103.7	0.0	106.1	106.1
Huesca	0.0	103.1	103.1	0.0	115.9	115.9
Huelva	1.4	100.8	102.2	4.1	125.6	129.7
Guadalajara	4.6	92.1	96.7	5.4	125.8	131.2
Teruel	5.2	91.2	96.4	5.6	110.7	116.3
Navarra	0.0	92	92.0	0.0	116	116
Albacete	4.5	87.2	91.7	9.0	113.5	122.5
Murcia	2.8	87.6	90.4	0.0	109.2	109.2
Soria	2.7	86.4	89.1	0.0	105	105
Pontevedra	0.0	89	89.0	0.0	106.8	106.8
Guipuzcoa	0.0	88.8	88.8	0.0	109.9	109.9
Oviedo	0.0	87.5	87.5	7.2	105.7	112.9
Castellon	0.0	84.7	84.7	0.0	108.3	108.3
Ciudad Real	6.4	70.2	76.6	10.5	86.4	96.9
Cuenca	4.6	70.2	74.8	0.0	75.9	75.9
Valencia	1.1	73.3	74.4	0.0	91.3	91.3
Barcelona	0.0	74.4	74.4	0.0	92.1	92.1
Jaen	7.9	65.8	73.7	8.3	73.9	82.2
Lerida	0.0	72.9	72.9	0.0	84.4	84.4
Baleares	0.0	72.8	72.8	0.0	83.2	83.2
Toledo	6.0	66.4	72.4	15.2	80	95.2
Tarragona	0.0	71.9	71.9	0.0	83.5	83.5
Badajoz	4.6	59.5	64.1	15.1	76.2	91.3
Gerona	0.0	62.9	62.9	0.0	73.2	73.2
Cadiz	5.6	54.7	60.3	9.3	79.3	88.6
Cordoba	8.7	49.6	58.3	19.9	57	76.9
Caceres	3.1	53.9	57.0	5.0	70.8	75.8
Madrid	10.3	37.3	47.6	11.7	55	66.7
Malaga	2.5	30.8	33.3	5.0	41	46
Sevilla	3.3	29.0	32.3	9.2	41	50.2
Canarias	0.0	6.1	6.1	0.0	6.2	6.2
Total Spain	2.4	92.6	95.1	3.0	112.9	115.9

Excess mortality estimates were based on a seasonal regression model applied to monthly mortality and presented as rates per 10,000. Provinces are sorted according to the cumulative excess pandemic respiratory mortality rates.

**Table 3:** Multivariate regression models of excess death rates associated with the spring, fall, and winter pandemic waves as a function of socio-demographic variables across provinces of Spain

**Spring wave**						
**Predictor**	**Coefficient**	**Standard error**	**P value**	**RMSE**	**Intercept**	**Predictor is in final model?**
**Baseline mortality rates**	0.884	0.073	<0.001	1.31	−0.7852	Yes
Latitude	−0.017	0.074	0.822			No
Longitude	−0.041	0.058	0.478			No
Population density	0.004	0.006	0.505			No
Population size	0.000	0.000	0.764			No
Proportion of school age children 5–15 years	7.336	9.547	0.446			No
Infant mortality rates	0.002	0.002	0.168			No
**Urbanization**	0.049	0.015	0.002			Yes
**Fall wave**						
**Predictor**	**Coefficient**	**Standard error**	**P value**	**RMSE**	**Intercept**	**Predictor is in final model?**
Baseline mortality rates	−0.68	1.55	0.67	29.8	−315.1	No
**Latitude**	7.02	1.61	<0.001			Yes
Longitude	0.21	1.76	0.90			No
**Population density**	−0.20	0.11	0.08			Yes
Population size	0.00	0.00	0.42			No
**Proportion of school age children 5–15 years**	403.45	205.47	0.06			Yes
Infant mortality rates	−0.02	0.03	0.60			No
Urbanization	−0.21	0.46	0.65			No
**Winter wave**						
**Predictor**	**Coefficient**	**Standard error**	**P value**	**RMSE**	**Intercept**	**Predictor is in final model?**
**Baseline mortality rates**	0.754	0.048	<0.001	1.86	−2.5	Yes
Latitude	−0.121	0.109	0.275			No
Longitude	−0.048	0.085	0.573			No
Population density	0.005	0.008	0.548			No
Population size	0.000	0.000	0.462			No
Proportion of school age children 5–15 years	−14.092	12.843	0.278			No
**Infant mortality rates**	0.005	0.002	0.014			Yes
Urbanization	0.009	0.022	0.699			No
**Cumulative excess rates**						
**Predictor**	**Coefficient**	**Standard error**	**P value**	**RMSE**	**Intercept**	**Predictor is in final model?**
Baseline mortality rates	−0.579	0.563	0.310			No
**Latitude**	7.082	1.528	<0.001			Yes
Longitude	0.050	1.674	0.976			No
**Population density**	−0.196	0.105	0.070			Yes
Population size	0.000	0.000	0.380			No
**Proportion of school age children 5–15 years**	395.934	195.292	0.049			Yes
Infant mortality rates	−0.001	0.033	0.972			No
Urbanization	−0.090	0.439	0.839			No

We generated simplified models by means of backward stepwise elimination. Predictors in final simplified models for each pandemic wave are highlighted in bold. Coefficients, standard errors, and P values for all other predictors correspond to the full regression model.

Spain experienced the main pandemic onslaught during August-December 1918, with respiratory mortality peaking on average in October 1918 and mean excess death rates estimated at 82.3 per 10,000 and 101.0 per 10,000 for respiratory and all-cause mortality, respectively (Table 2 and Figure 3). All provinces experienced substantial excess respiratory death rates during the fall pandemic wave in 1918, except for the Canary Islands (5.4 per 10,000 people) and low excess respiratory mortality levels in the southern provinces of Sevilla (29 per 10,000) and Malaga (30.8 per 10,000, Figures 3 and 4). In contrast, the province of Burgos experienced the highest excess death rate during the fall wave at 167.7 and 212 per 10,000 people based on respiratory and all-cause mortality, respectively (Table 2 and Figure 4). Excess mortality rates derived from respiratory diseases and all causes were strongly correlated (Spearman rho = 0.98, P < 0.001) However, the province with highest relative risk (RR) of pandemic excess mortality was Palencia in northwest Spain, with ~210% mortality elevation over baseline respiratory mortality followed by Burgos (200%) and Alicante (170%) (Additional file 1: Figure S2). During the fall pandemic wave, 37.9% of the variability in excess respiratory death rates across provinces was explained by a model that included latitude (P = 0.0001), population density (P = 0.08), and the proportion of children living in the population (P = 0.056) (Table 3). Significant spatial autocorrelation in excess respiratory death rates was identified during the fall 1918 wave (Moran’s I test, P = 0.03).

Fifty seven percent (28/49) of the Spanish provinces experienced excess respiratory death rates during the winter 1919 (January-April 1919), with excess respiratory death rates during winter 1919 ranging from 0 to 20.9 deaths per 10,000 (Table 2). Moreover, excess death rates calculated from respiratory and all-cause mortality were strongly correlated during this pandemic wave (Spearman rho = 0.82, P < 0.001). We did not find significant correlations between excess mortality rates in the spring, fall or winter pandemic waves (P > 0.06). There was also significant spatial autocorrelation in excess respiratory death rates during the winter 1919 (Moran’s I test, P = 0.015; Figure 4). A model that included baseline mortality rates (positive association; P < 0.0001) and infant mortality rates (positive association; P = 0.01) explained 84.4% of the variability in excess respiratory death rates during the winter 1919 (Table 3).

Cumulative excess deaths from May 1918 to April 1919 were estimated at 194,960 (95.1 per 10,000) and 237,600 (115.9 per 10,000) for Spain based on respiratory and all-cause mortality, respectively (Table 2). Cumulative excess pandemic respiratory death rates ranged from 6.1 per 10,000 for the Canary Islands to 169.7 per 10,000 for Burgos, respectively (Figure 4). There was significant spatial autocorrelation in cumulative excess respiratory death rates (Moran’s I test, P = 0.03), with a number of provinces in northwest Spain experiencing the highest excess respiratory mortality rates. Stepwise regression identified latitude (P < 0.0001), density (P = 0.07), and proportion of children (P = 0.05) as predictors of cumulative excess respiratory death rate (variance explained 40.4%, Table 3).

## Discussion

We have conducted a detailed spatial-temporal analysis of the 1918 influenza pandemic in 49 Spanish provinces by modeling monthly time series of respiratory and all-cause mortality covering during the period January-1915 to June-1919. Our analysis revealed heterogeneous geographic patterns and substantial pandemic mortality impact in most provinces. We identified 3 pandemic waves of varying timing and intensity covering the period from May 1918 to April 1919, with highest pandemic-related excess mortality rates occurring during the months of October-November across all Spanish provinces. Northwestern provinces were among the most affected (110.2-169.7 excess respiratory deaths per 10,000) as well as Almeria (169.1 per 10,000) in the south. A model that included latitude, population density, and the proportion of children explained about 40% of the variability in cumulative excess death rates.

The first pandemic wave generated a relatively mild impact in Spain with excess respiratory mortality rates ranging from 0 to 10.3 excess deaths per 10,000 during May-July 1918, affecting about half of the Spanish provinces. Pandemic activity was concentrated in Central Spain, particularly in Madrid, although excess mortality was also identified more sporadically in southern and northern provinces. The earliest news report on the appearance of the 1918 influenza pandemic in Spain was in the Madrid newspaper ‘El Sol’ on 22 May 1918 [4,10] at a time when pandemic-related news were censored among countries participating in World War I. It is also worth mentioning that about 68% of the population of Madrid lived in unsanitary conditions in 1918 [38]. The early onset, high mortality rate, and news reports associated with the spring pandemic wave in Madrid may have contributed to the fact that today the 1918 influenza pandemic is known as the “Spanish flu” or the “Spanish Lady”.

It is likely that the virus reached Spain by train via temporary Spanish workers who traveled to and from France due to the shortage of young French workers [3]. Of note, earlier respiratory disease outbreaks associated with the pandemic had been reported in France in April 1918 [2,10]. The possibility that the virus could have been introduced into Spain from the southern Spanish border cannot be ruled out although the earliest reports of pandemic influenza in The Strait of Gibraltar did not occur until May 1918, a month after the respiratory disease reports in France [2]. Early pandemic waves associated with low excess mortality rates have been documented in other regions during February-July 1918 including New York City [5], Mexico [7], Geneva [39], Copenhagen [19], the US military [40], the UK [23], and Singapore [11]. These epidemiological findings are in line with virologic evidence of pandemic A/H1N1 influenza infection among US soldiers in May 1918 [41].

We did not detect any measurable pandemic mortality impact in respiratory or all-cause data during May-July 1918 in both the Canary and Balearic Islands (Table 2). This suggests that the pandemic virus was not introduced into these Spanish islands until fall 1918, probably due to the dramatic decline in maritime traffic associated with the closing of European markets and the threat of German submarines during World War I. However, important underreporting of births and deaths during 1900–1930 in the Canary Islands has been noticed [42], and therefore we cannot discard the possibility that pandemic influenza outbreaks could have occurred unreported in Spanish islands in spring-summer 1918. Alternatively, these Spanish islands could have experienced low-mortality waves, which could be difficult to detect from mortality statistics alone. For instance, the herald pandemic wave of 1918 in Denmark was only clearly evident from morbidity data [19].

Spain exhibited high excess respiratory mortality rates during the fall pandemic wave in 1918 except for the Canary Islands, located in the Atlantic in front of the African coast of the Sahara, with an excess death rate estimated at 5.4 per 10,000. We hypothesize that climatological conditions and specific population characteristics of the Canary Islands limited the transmission and severity of the pandemic virus in these populations. In contrast, the excess respiratory mortality burden in the Balearic Islands was substantially higher at 68.1 per 10,000, an estimate that is more in line with those of the provinces of Valencia or Castellon (71.0 and 84.7 per 10,000), which are located at about the same latitude as the Balearic Islands. We note that high mortality rates reported in island populations have been reported for remote Pacific islands [43], and there is very little data on island mortality rates in other regions, including Europe. The huge mortality rates reported in Pacific islands could be driven by risk factors specific to the aboriginal populations whereas indigenous populations of the Canary Islands were decimated by the Spanish in the 15th century.

The onset of the fall pandemic wave in 1918 coincided with the nationwide celebration of traditional holidays at the end of the summer [3,28,38,44] and with the recruitment of soldiers in September [3]. This lethal second wave generated higher excess mortality rates in northern provinces than in southern provinces, in provinces with higher population density, and those with higher proportion of children. However, over 50% of the variability in excess respiratory mortality remained unexplained which indicates that other unidentified factors (e.g., climate, background immunity) could have played a role. These results resemble the geographically heterogeneous pandemic mortality patterns of the 2009 A/H1N1 influenza pandemic. Indeed, differences in background death rates do not align with heterogeneity in 2009 pandemic mortality outcomes across countries [45].

Spain experienced a substantial recrudescent wave of respiratory pandemic mortality in winter 1919. It is worth noting that Madrid was the only Spanish province that exhibited a protracted fall-winter pandemic wave as shown in Figure 1. This pattern suggests a slower pandemic growth rate and lower reproduction number during this period in Madrid compared to other provinces, which probably resulted from a substantial reduction in susceptibility levels in the capital city resulting from the earlier spring-summer wave [19,40]. The variability in excess mortality rates across affected provinces in winter 1919 was partly explained by baseline mortality and infant mortality rates, which suggests that infants <1 year were significantly affected during the third wave of the pandemic [3].

Spain experienced some of the highest excess mortality rates during the 1918–1919 influenza pandemic in Europe [18] despite the fact that this country did not take part in World War I. Perhaps this is not surprising as Spain was going through a demographic transition with elevated mortality rates that were only comparable to those of Eastern Europe. Of note, the life expectancy in Spain in 1910 was 41 years and declined to 40 in 1920 as a result of the pandemic impact [32]. In our analysis we found that latitude, population density, and the proportion of children living in provinces explained about 40% of the variability in cumulative excess death rates across provinces in Spain during the study period.

Cumulative excess mortality rates followed a South–north gradient after controlling for all other demographic factors, with northern provinces generally experiencing the highest excess mortality rates. Experimental studies indicate that influenza transmission is favored by lower temperatures and humidity levels (e.g., [46]), and we speculate that more favorable climate conditions in northern Spain could partially explain this pattern [47,48]. In particular, southern provinces experience higher temperatures than northern provinces. By contrast, a North–South gradient in excess mortality burden associated with the 1918 influenza pandemic has been reported at a broader spatial scale across Europe, a pattern that was likely shaped by socio-economic conditions [18]. While socio-economic conditions, climate factors, and background immunity, may all contribute to driving influenza excess mortality rates, the relative contribution of each factor remains debated and could depend on the spatial scale of the study.

Our study has several limitations. First, our data were retrieved from monthly mortality statistical bulletins issued by the Spanish government, but these bulletins were not consistently available after June 1919, which precluded the estimation of the excess mortality during subsequent pandemic waves. Second, our mortality time series were not stratified by age or gender which would have been useful to monitor demographic shifts throughout the pandemic. A previous study by Erkoreka [10] analyzed the proportional distribution of influenza death counts in Madrid prior and reported a shift in the proportional distribution of influenza deaths from older populations (> = 65 yrs.) prior to the 1918 pandemic to young adults (15–34 yrs.) during the 1918 pandemic waves [49]. Then this age pattern reverses to the characteristic profile of seasonal influenza by 1921 [10,50]. Finally, we assumed that the infant mortality rate was a reasonable proxy for health index as in prior studies [23]. However, we speculate that infant mortality was strongly seasonal at the time and could be highest in summer months.

## Conclusion

In conclusion, our spatial-temporal analysis of excess respiratory mortality rates during the 1918 influenza pandemic in Spain reveals a dramatic and heterogeneous mortality burden associated with this pandemic. This is the first geographically comprehensive study of the epidemiology of the pandemic in Spain, a particularly heavily affected European country which will forever remain associated with the pandemic through the qualifier of “Spanish Influenza”. Our findings suggest that a combination of local factors including variation in baseline mortality rates, population density, urbanization, infant mortality rates, age population structure, infant mortality rates, and climatic conditions modulated the spatial-temporal evolution and mortality burden associated with the 1918 influenza pandemic. Further research could concentrate on regions where, in addition to detailed mortality data, other type of information is available, including the prevalence of underlying risk factors or individual-level patient data. We are still a long way from fully understanding the determinants of mortality associated with the most devastating influenza pandemic in recorded history.

## Competing interests

The authors declare that they have no competing interests.

## Authors’ contributions

GC, AE, CV, and BE designed the study. BE participated in data acquisition. GC analyzed the data and wrote the first draft of the manuscript. GC, AE, CV, and BE participated in the interpretation of results. All authors contributed to the writing and editing of the manuscript. All authors read and approved the final manuscript.

## Pre-publication history

The pre-publication history for this paper can be accessed here:

http://www.biomedcentral.com/1471-2334/14/371/prepub

## Supplementary Material

Additional file 1: Figure S1 Relative risk of death over the mortality baseline across provinces of Spain according to pandemic periods. Figure S2 Relative risk ratio of respiratory mortality across provinces of Spain. Click here for file
