# Supplementary material for: Spatial-temporal excess mortality patterns of the 1918–1919 influenza pandemic in Spain
Source: BMC Infect Dis. 2014 Jul 5;14:371. doi: 10.1186/1471-2334-14-371 (PMC4094406; doi:10.1186/1471-2334-14-371)
Supplement: Additional file 1: Figure S1 — Relative risk of death over the mortality baseline across provinces of Spain according to pandemic periods. Figure S2 Relative risk ratio of respiratory mortality across provinces of Spain. [file 1471-2334-14-371-S1.docx]

**Figure S1.** Relative risk of death over the mortality baseline across provinces of Spain according to pandemic periods (May-July 1918, August 1918-December 1918, and January 1919-April 1919) and sorted from high to low cumulative relative risk of death. The relative risk of death is based on the ratio of excess mortality to background mortality, facilitating comparisons across provinces with different background risks of death.

**
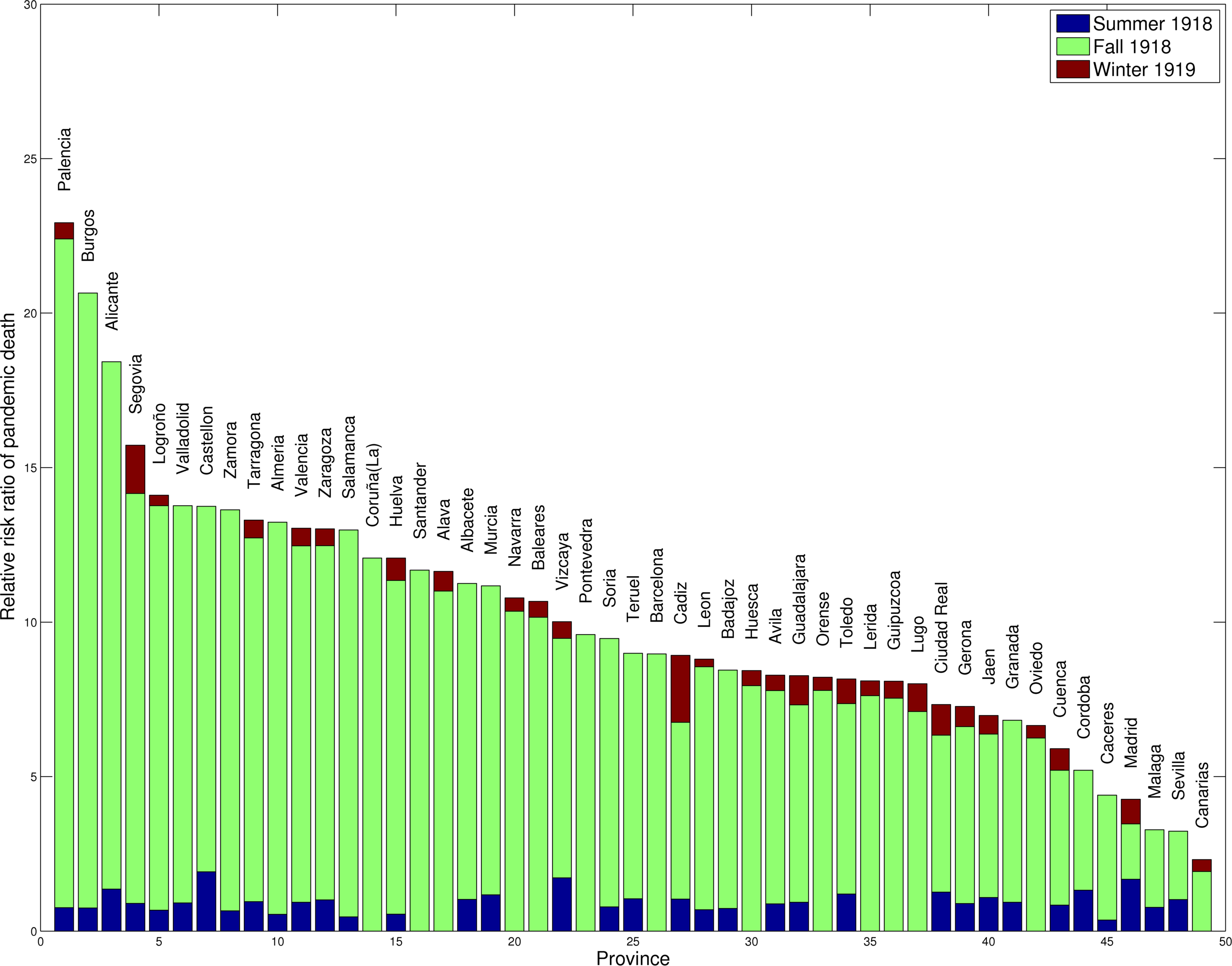
**

**Figure S2**. Relative risk ratio of respiratory mortality across provinces of Spain during the spring (May 1918-July 1918), fall (August 1918-December 1918), and winter (January 1919-April 1919) pandemic waves and the cumulative relative risk ratio associated with the 1918-1919 influenza pandemic.

| 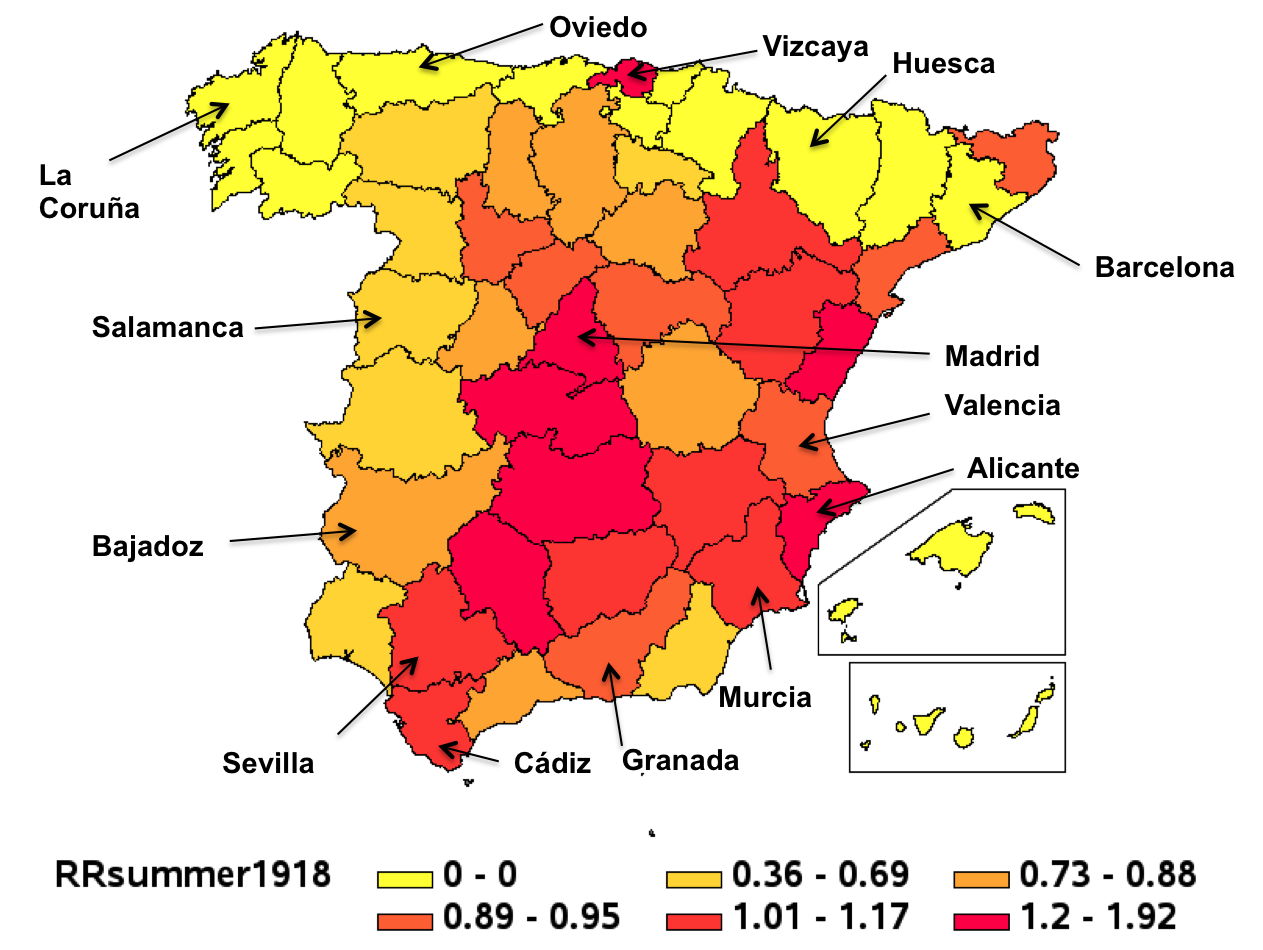 | 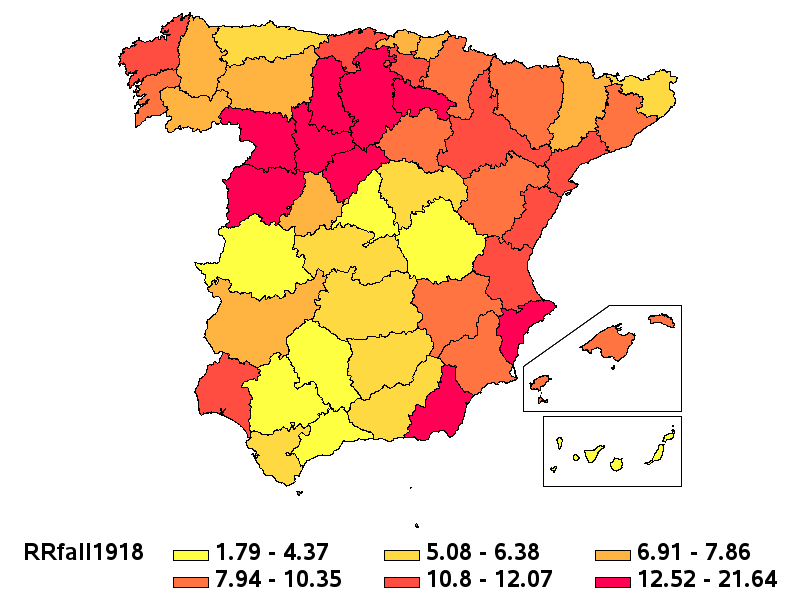 |
| --- | --- |
| 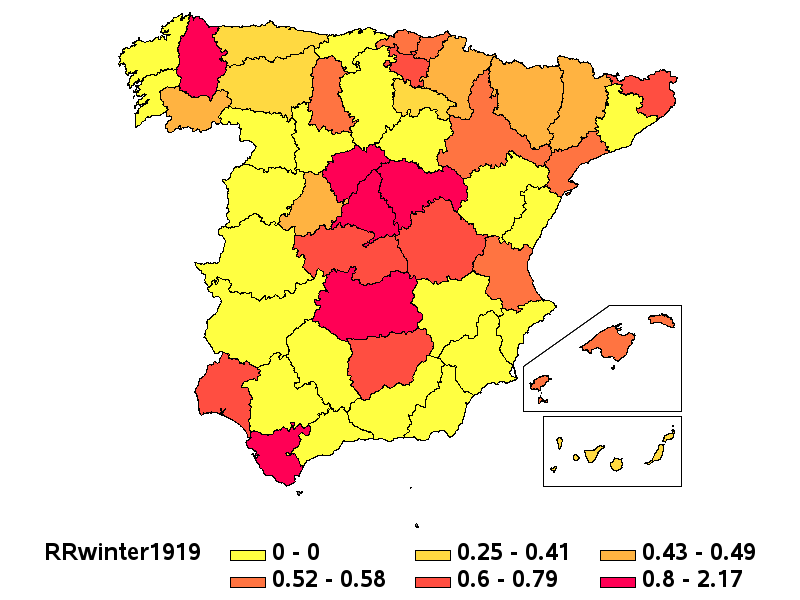 | 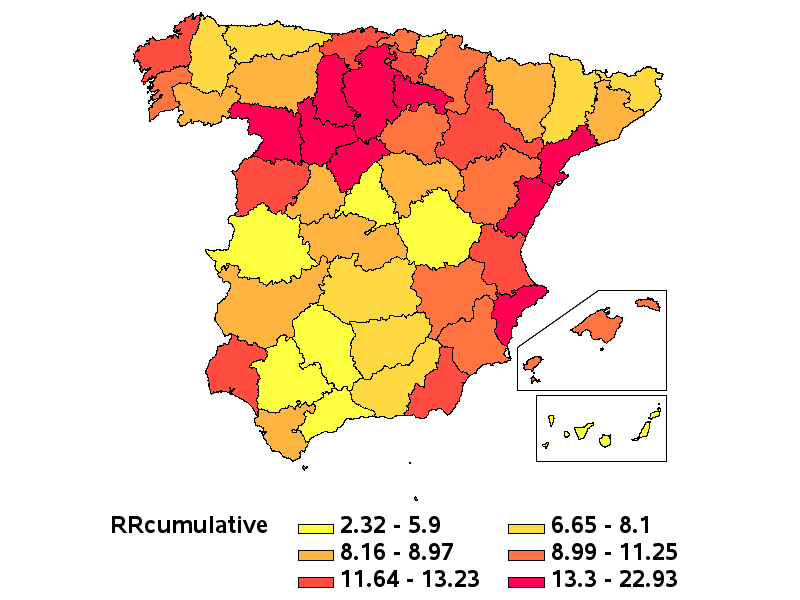 |
